# Survival from prostate cancer in England and Wales up to 2001

**DOI:** 10.1038/sj.bjc.6604596

**Published:** 2008-09-23

**Authors:** N W Clarke

**Affiliations:** 1 Department of Urological Oncology, Christie Hospital NHS Trust, Wilmslow Road, Manchester M20 4BX, UK

On superficial analysis, the results presented by Rowan *et al* (2008) might suggest that there is a cause for celebration. Surely a major increase in survival in a disease, which is the biggest killer of men in the United Kingdom, shows that diagnosis and treatment is getting better? Before we get too elated, however, we should perhaps pause for thought, for as the authors rightly point out, there are important reasons why the results appear to be so good.

The data confirm that for the United Kingdom, like other developed countries, diagnostic rates for prostate cancer have risen sharply in the last two decades. This phenomenon has emerged outside the United Kingdom as a consequence of population-based screening using the serum PSA test (Merrill and Stephenson, 2000) and although this type of systematic cancer testing is not carried out in the United Kingdom, it is well known that opportunistic PSA testing has become widespread and that there is considerable ‘case finding’ in asymptomatic men as a consequence (Melia *et al*, 2004; Gavin *et al*, 2004). It is therefore no surprise to see that the rate of diagnosis in the United Kingdom is going up, as has been shown in this study. It is also well known that this epidemiological trend has lead to a ‘stage shift’, with diagnosis and treatment occurring earlier in the natural history of the disease in many men (Andriole *et al*, 2006).

In interpreting evolving epidemiological trends in this area, it is also important to consider the underlying change in mortality. Clearly, there are potential errors inherent in correlating relative survival (the interval between diagnosis and all causes of death) with the prostate cancer-specific death rate (cause of death as recorded on a death certificate) and it would be inappropriate to correlate the changes in survival and mortality too rigidly. Nonetheless, it is important to note that although prostate cancer-related mortality has decreased slightly in the United Kingdom and in other parts of the developed world over this time period, it has not changed fundamentally (Cancer Research UK, 2007). It is also important to consider the well-described lead-time effect (the time difference between screen-based detection and clinical detection in the absence of screening) in prostate cancer, which is relevant in this population and has been estimated to be in the region of 10 years or more (Draisma *et al*, 2003). It is possible that this and other phenomena may have a significant bearing on the changes observed in this report.

The effects of treatment on this population are difficult to quantify. The authors largely discount any treatment effect in their analysis, although it is not possible, on the basis of the information presented, to determine whether a therapeutic component has contributed in some way to the observed survival improvements. The explicit statement that there has been no ‘substantially improved treatment’ for prostate cancer is not strictly true. There is level 1 evidence to show that surgical treatment has a beneficial effect in terms of absolute survival in localised disease (Bill-Axelson *et al*, 2005) and in treatment schemes using combined adjuvant hormone and radiation therapy for high-risk locally advanced disease Bolla *et al*, 2002). Interventions in late-stage disease are also much more widespread (Khafagy *et al*, 2007). Thus, therapeutic intervention may well have made a contribution to the observed effect in the patient population studied. That being said, it is likely that this effect would have been relatively small and that other factors, such as lead time rather than specific treatment intervention, have been the major contributory factors to the dramatic changes documented.

The results in relation to different socioeconomic groups are of interest. In considering the ‘difference in survival’ in the affluent and deprived groups it is again critical to look at the differential rates of diagnosis. If the number of low-risk ‘screening type’ cancers is lower in the less affluent group, then their survival relative to that in the more affluent groups may seem worse. There are reasons why the diagnostic rate may be much lower in the deprived groups. Deprived populations are known to have a lower rate of uptake for early diagnostic techniques such as PSA testing (Wannamethee and Shaper, 1997) and this is borne out by evidence that the presentation of men with prostate cancer has not changed over a number of years in some deprived populations, notwithstanding the advent of PSA testing (Mokete *et al*, 2005). Rowan *et al* (2008) suggest that affluent men may have ‘greater access’ to PSA tests. There is some evidence that this may be true; Mokete *et al* (2005) also showed that the rate of testing may be lower by a factor of almost 50% in deprived communities, although it is not clear whether this is owing to lack of availability of the test from General Practitioners or whether men in this particular social group simply do not come forward for testing.

But has this disadvantaged the socially deprived male? It is interesting to reflect that they, as a group, may have been spared the physical and psychological effects of overtreatment, which are currently problematic in early prostate cancer in some countries (Carroll, 2005) and furthermore, the type of tumour more likely to have been detected would have been low-volume/low-risk prostate cancer (Parker *et al*, 2006) with a good prognosis in the long term (Albertson *et al*, 2005). In this sense, the socially deprived male may, for once, have emerged better off than his affluent counterpart. However, other men in this group, whose cancers represent a real threat to their lives will have been disadvantaged.

Overall, the point remains that improvements in survival patterns are encouraging but lead time and early diagnosis of greater numbers of patients with low-stage/low-risk prostate cancer may have a significant bearing on this. It is fundamentally important to emphasise that the overall death rate is a critically important figure and to remember that this disease is still responsible for one cancer death in eight in men in the United Kingdom. There is still no effective curative treatment for clinically aggressive prostate cancer. Recently, an analysis comparing SEER data with UK Hospital Episode Statistics (HES) has shown a relatively static picture in the United Kingdom compared with more rapid improvements in absolute death rates, which may have resulted from more aggressive treatment policies in the United States for both early and late disease (Collin *et al*, 2008).
